# Eat Slowly

**Published:** 1895-02

**Authors:** 


EAT SLOWLY.—The importance of eating slowly is so great that children should be trained to this; no matter how hungry they are, insist upon the food being thoroughly masticated. A small quantity of food well ground will prove much more wholesome and nourishing than a hearty meal hastily swallowed. Cold food is more difficult to digest than hot, if eaten in too great haste. Before digestion takes place the food has to be raised to the normal temperature of the stomach, which is about ninety-eight degrees.

